# STAT6 contributes to renal fibrosis by modulating PPARα-mediated tubular fatty acid oxidation

**DOI:** 10.1038/s41419-022-04515-3

**Published:** 2022-01-19

**Authors:** Jianzhong Li, Youjing Yang, Qianmin Li, Shuhui Wei, Yujia Zhou, Wangjianfei Yu, Lian Xue, Ling Zhou, Lei Shen, Guoyuan Lu, Ling Chen, Shasha Tao

**Affiliations:** 1 Medical College of Soochow University, 199 Ren’ai Road, Suzhou, 215123 China; 2 Department of Nephrology, The First Affiliated Hospital of Soochow University, Suzhou, Jiangsu 215006 China; 3 School of Public Health, Medical College of Soochow University, 199 Ren’ai Road, Suzhou, 215123 China

**Keywords:** Lipid signalling, Medical research

## Abstract

Lipid metabolism, especially fatty acid oxidation (FAO) dysfunction, is a major driver of renal fibrosis; however, the detailed regulatory mechanisms involved remain unclear. In this study, we showed that there existed an association between the signal transducer and activator of transcription 6 (STAT6) and tubular lipid metabolism in fibrotic kidneys. Specifically, STAT6 was activated along with the accumulation of lipids via the downregulation of FAO-related genes when mice were subjected to unilateral ureteral obstruction (UUO) or high-fat diet challenge. Tubular-specific depletion, or pharmacologic inhibitor of Stat6 in mice, and Stat6 knockdown in cultured tubular cells attenuated lipid accumulation and renal fibrosis by enhancing FAO. Mechanistically, STAT6 transcriptionally inhibited the expression of PPARα and its FAO-related target genes through a sis-inducible element located in the promoter region of the protein. In conclusion, our study demonstrates the mechanistic details of STAT6-mediated FAO dysregulation in the progression of renal fibrosis and provides a preclinical rationale for efforts to improve the management of renal fibrosis brought about by FAO dysregulation.

## Introduction

Chronic kidney disease (CKD) is an important public health issue with enormous social and economic consequences [1, 2]. Renal fibrosis, which is manifested by tubular atrophy and excessive extracellular matrix (ECM) accumulation, is the most prominent hallmark of CKD; it is caused by kidney damage and is closely associated with the progression and prognosis of the disease [3, 4]. Given the potential prevalence and poor prognosis of renal fibrosis, understanding the mechanisms leading to this condition is an urgent priority.

Tubular cells are highly specialized cells with a high demand for adenosine triphosphate (ATP) and primarily depend on fatty acid oxidation (FAO). Emerging research suggests that impaired FAO in tubular cells and the consequent onset of intracellular lipid accumulation have a profound impact on the fate of tubular cells. Indeed, impaired FAO elicits epithelial–mesenchymal transition (EMT), inflammation, and eventually interstitial fibrosis [5–7]. Peroxisome proliferator-activated receptor α (PPARα) is a key ligand-activated transcription factor widely considered to be a master regulator of mitochondrial FAO activity and triglyceride (TG) catabolism [8–10]. Earlier studies indicated that defective PPARα could reduce mitochondrial FAO and exacerbate kidney fibrosis [11, 12].

Signal transducer and activator of transcription 6 (STAT6) acts as a Th2-type cytokine-based transcriptional activator that is abundantly expressed in multiple tissues and organs. Previous studies have demonstrated its pleiotropic effects, including its participation in macrophage polarization, cancer progression [13–15], human oncogenic herpesvirus activation [16], microglia/macrophage efferocytosis, and stroke improvement [17]. However, the role and the detailed mechanisms of tubular STAT6 in renal fibrosis remain unclear. Our previous study revealed that high STAT6 expression could be observed in the renal tubular cells of fibrotic kidneys and that transfection of STAT6 in tubular cells is critical for TGF-β and fibrotic marker expression [18]. Such results indicate the possible regulation of tubular STAT6 in renal fibrosis. Moreover, a previous report showed increases in the expression of PPARα and its target FAO-related genes in primary hepatocytes with STAT6 deficiency [19]. These findings indicate the possible regulation of STAT6 and FAO in renal fibrosis.

In this study, we provide strong in vivo and in vitro evidence demonstrating that activation of tubular STAT6 contributes to lipid accumulation and renal fibrosis by inhibiting FAO in the UUO model. STAT6 transcriptionally inhibited PPARα and its FAO-related targets through a sis-inducible element located in the promoter region of the protein.

## Results

### STAT6 signaling is activated in mice fibrotic kidneys

**Fig. 1:**
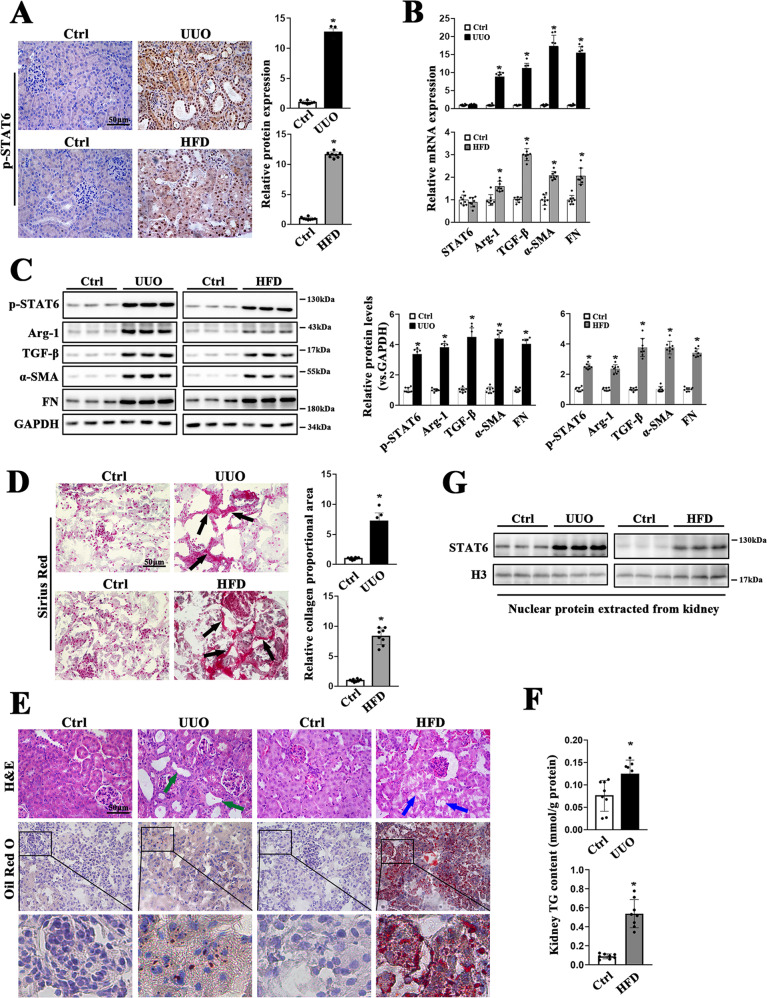
STAT6 is activated in the kidneys of UUO and HFD mice. **A** Representative micrographs for IHC staining of p-STAT6 in kidney section and quantification are performed. **B** The mRNA and **C** protein expression of STAT6, p-STAT6, Arg-1, TGF-β, α-SMA, FN in the indicated groups were determined by qRT-PCR or immunoblot analyses with the quantification on the right panel. **D** Representative micrographs for Sirius red and relative collagen proportion was quantified. **E** Representative micrographs for H&E and Oil Red O staining in kidney sections from indicated group. **F** Kidney TG content were measured in the indicated groups. Results are expressed as the mean ± SD (*n* = 8, *p < 0.05, Ctrl *vs*. Treatments). **G** Nuclear proteins were extracted from kidneys from different individuals as indicated, STAT6 and H3 were assayed by western blot analysis.

To investigate the possible association between STAT6 signaling and renal fibrosis, UUO and high-fat diet (HFD) mice models were employed (Fig. S1A). The obvious physiological and metabolic parameters changes were presented in HFD mice, but no change in UUO mice (Fig. S1B-K). In the kidney of the above models, STAT6 activation was first detected. Both the levels of STAT6 phosphorylation and its targets (Arg-1, TGF-β) expression were increased relative to the corresponding control along with the induction of α-SMA and FN (Fig. 1A–C). IHC staining showed that p-STAT6 positive staining mainly focused on tubular cells (Fig. 1A). Consistently, the immunoblot analysis of nuclear protein extracted from kidney tissue showed that the location of STAT6 was mostly in the nucleus (Fig. 1G). Furthermore, Sirius staining showed that obvious collagen deposition existed in the kidneys (Fig. 1D). Besides that, lipid accumulation was induced by these models as demonstrated by Oil Red O staining; kidney TG content was higher in model groups compared with the control group (Fig. 1E, F). To further confirm the association of STAT6 activation, lipid accumulation, and kidney fibrosis. Ischemia-reperfusion (I/R) and Aristolochic acid nephropathy (AAN) models were also employed (S2). Consistently, in the above models, the activation of STAT6 signaling pathway in renal tubular cells was accompanied by increased protein expression of p-STAT6, Arg-1, and TGF-β, along with the upregulation of α-SMA and FN (Fig. S2A, B). Sirius staining indicated obvious collagen deposition in the kidneys, and Oil Red O staining showed the accumulation of lipid in both IR and ANN kidney tissue (Fig. S2C, D); besides, kidney TG content was higher in these two model groups compared with the control group (Fig. S2E). Taken together, these simultaneous upregulation of STAT6 activation were observed in the mice with multiple renal fibrotic models, indicating the potential involvement of STAT6 in lipid metabolism and renal fibrosis.

Next, the RNA-sequencing dataset (GSE145053) retrieved from the Gene Expression Omnibus (GEO) database was analyzed to further investigate the involvement of STAT6 in an unbiased manner. Volcano plots identified 1682 RNA species that exhibited significant changes between control and UUO groups. Hierarchical clustering and ingenuity biological function analysis for 50 most highly upregulated and downregulated genes revealed that gene ontology terms associated with “fibrosis-related processes” and “metabolic processes” were notably changed (Fig. S1M–O). Then the differential expressed genes were clustered by MCODE (Cytoscape). The PPI network including these two clusters was assessed and STAT6 was identified as potentially relevant with tissue fibrosis and PPARα-associated fatty acid metabolism (Fig. S1P–Q). All the evidence above suggested STAT6 signaling, especially that in the tubular cells, was implicated in the process of lipid metabolism and kidney fibrosis.

### Ablation of STAT6 in tubular cells attenuates renal fibrosis

**Fig. 2:**
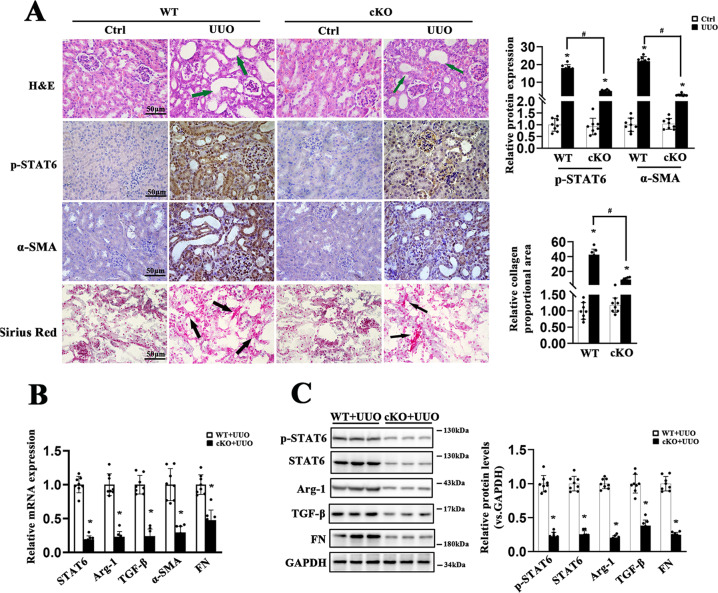
STAT6 deficiency in tubular cells attenuates UUO-caused renal fibrosis. The tubular-specific-Stat6 KO and control littermates were sacrificed 1w after UUO operation. **A** Representative IHC staining of p-STAT6, α-SMA, and micrographs for H&E, Sirius red staining in the control and fibrotic kidneys from the indicated group and quantification was performed. **B**The mRNA and **C** protein expression of p-STAT6, STAT6, Arg-1, TGF-β, α-SMA, FN in the indicated treatments were determined by qRT-PCR or immunoblot analyses with the quantification on the right panel. Results are expressed as the mean ± SD (*n* = 8, *p < 0.05, Stat6 WT *vs*. Stat6 cKO).

To further explore the potential role of tubular STAT6 signaling in regulating kidney fibrosis and lipid metabolism, renal tubular-specific-Stat6 KO mice were generated by crossbreeding Stat6^flox/flox^ and γGT^Cre^ mice (Figs. 2A, S3A). No significant differences existed in body weight (BW), kidney weight (KW), and the ratio of KW to BW (KW/BW) between WT and Stat6 cKO mice (Fig. S3B–D). Blockage of STAT6 signaling was observed in the renal tubular cells of cKO mice compared to the WT mice through detecting the mRNA and protein abundance of STAT6, p-STAT6, and its target Arg-1 with or without UUO performance (Figs. 2A–C, S3A). Additionally, H&E and Sirius red staining indicated that collagen deposition and tubular atrophy caused by UUO were largely alleviated in Stat6 cKO mice, which was consistent with the protein and mRNA abundance of α-SMA, FN, and TGF-β (Fig. 2A–C). Taken together, STAT6 deficiency in tubular cells alleviates tubulointerstitial fibrosis.

### Ablation of STAT6 in tubular cells blocks renal lipid accumulation with increased FAO

**Fig. 3:**
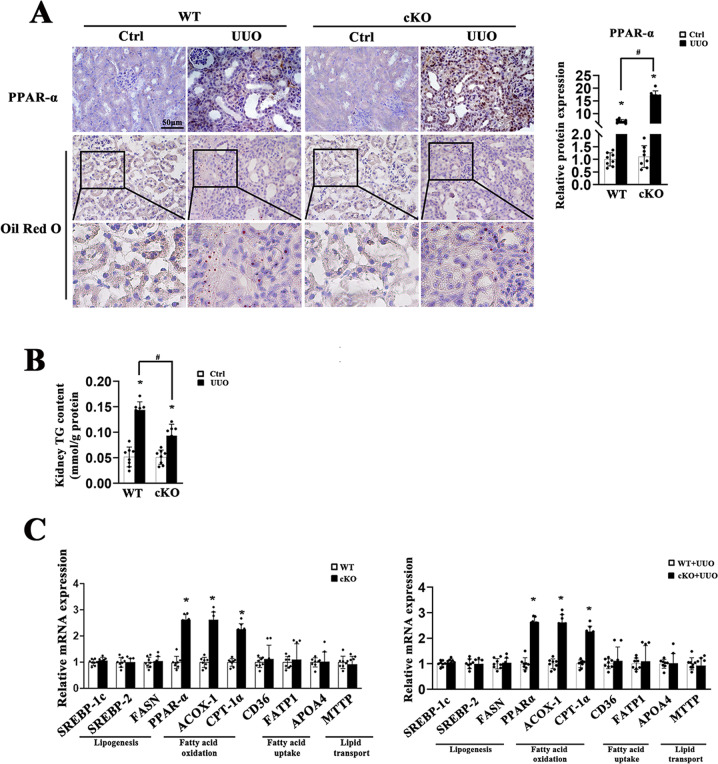
STAT6 deficiency in tubular cells attenuates UUO-caused lipid accumulation. The tubular-specific-Stat6 KO and control littermates were sacrificed 1w after UUO operation. **A** Representative IHC staining of PPARα and Oil Red O staining from Stat6 WT and cKO kidneys with or without UUO, and quantification for PPARα was performed. **B** TG content were determined in the kidneys from Stat6 WT and cKO mice with or without UUO. **C** mRNA levels of genes related to lipid metabolism in Stat6 WT and cKO mice with or without UUO operation. Results are expressed as the mean ± SD (*n* = 8, *p < 0.05, Stat6 WT *vs*. Stat6 cKO).

We further examined changes in lipid metabolism between the WT and Stat6 cKO mice. lipid accumulation and TG content in fibrotic kidneys from cKO UUO-treated mice were also blunted when compared to kidneys from WT UUO-treated mice, no significant difference of physiological parameters was observed between the contralateral groups (Fig. 3A, B). Additionally, Stat6 cKO mice exhibited higher expression of FAO-related genes, such as PPARα, ACOX-1 (acetyl-CoA oxidase 1), CPT-1α (carnitine palmitoyltransferase-1α) rather than genes related to lipogenesis, fatty acid uptake, and lipid transport with or without UUO performance (Fig. 3C). The results were consistent with the IHC staining for PPARα of kidney tissue, especially in tubular cells (Fig. 3A). Thus, in addition to less renal fibrosis, mice with renal tubular-specific-Stat6 KO showed reduction in lipid accumulation with increased FAO.

### STAT6 regulates PPARα signaling-mediated lipid metabolism in tubular cells

**Fig. 4:**
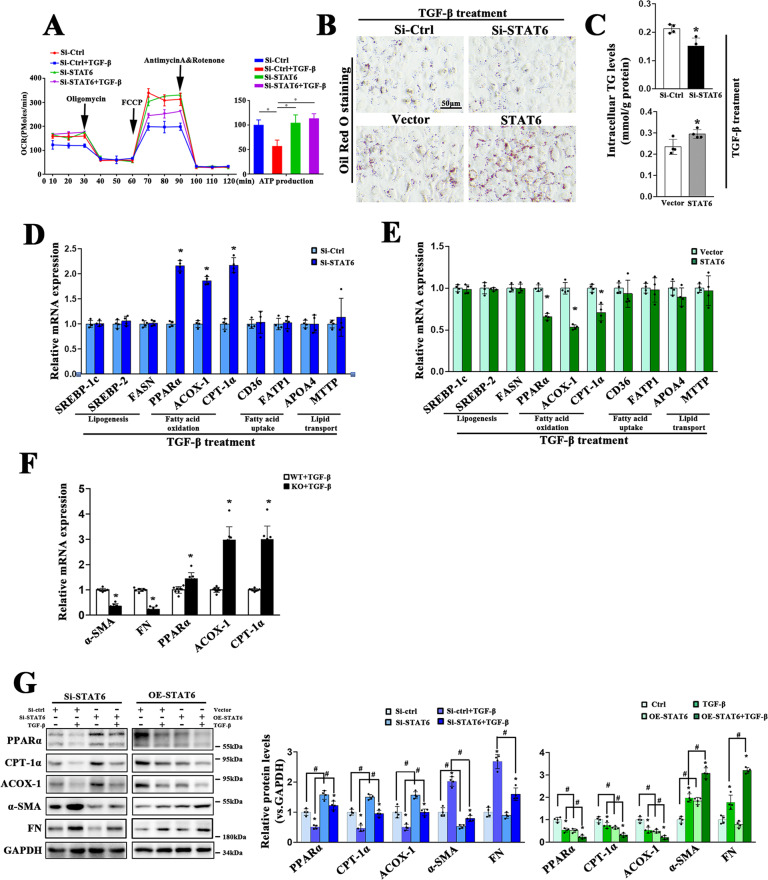
STAT6 in tubular cells suppresses PPARα-mediated fatty acid oxidation. HK2 cells were transfected with siRNA or plasmid for STAT6 inhibition or overexpression. Followed by 24 h serum-free medium culture, the cells were treated with TGF-β (5 ng/ml) for another 24 h. **A** Representative traces of three independent experiments for the measurement of oxygen consumption rate (OCR) were shown. Oligomycin, FCCP, and antimycin/rotenone were administrated at the indicated time point. ATP production was determined according to the OCR values. Data are presented as mean ± SD (*n* = 3, *P < 0.05). **B** Representative micrographs for Oil Red O staining of HK2 cells treated as indicated (**C**) TG content was determined enzymatically. **D**, **E** mRNA levels of genes related to lipid metabolism in STAT6 inhibited or overexpressed HK2 cells were determined by qRT-PCR. Results are expressed as the mean ± SD (*n* = 4 *p < 0.05, SiCtrl/Vector *vs*. SiSTAT6/STAT6 transfection) **F** mRNA levels of genes related to FAO and fibrotic proteins expression in primary renal tubular epithelial cells isolated from the above Stat6 WT and cKO mice. Results are expressed as the mean ± SD (*n* = 4, *p < 0.05, Stat6 WT *vs*. Stat6 cKO). **G** HK2 cells were transfected with indicated siRNA or plasmid for 24 h in serum-free medium and followed by TGF-β (5 ng/ml) treatment for another 24 h. Cell lysates were harvested and subjected to immunoblot analyses with the indicated antibodies. Quantification of relative protein expression was determined. Results are expressed as the mean ± SD (*n* = 4, *p < 0.05, Ctrl *vs*. TGF-β; ^#^*p* < 0.05, SiCtrl/Vector *vs*.SiSTAT6/STAT6 transfection).

To explore whether STAT6 regulates kidney fibrosis through modulating lipid metabolism, the effects of STAT6 on lipid metabolism were determined in vitro. Mitochondrial oxygen consumption rates (OCR) were first assessed by a seahorse analyzer. HK2 cells were transfected with control or STAT6 siRNA followed by TGF-β treatment. The results showed that OCR was markedly blocked after TGF-β treatment, and this effect was reserved by STAT6 downregulation, along with higher intracellular ATP produced by FAO compared with the control group (Fig. 4A). TG contents and lipid accumulation were attenuated by STAT6 siRNA, while enhanced by STAT6 overexpression with TGF-β treatment for 24 h (Figs. 4B, C, S4A), but not under the physiological condition (Fig. S4B), which was consistent with our in vivo data above. Additionally, STAT6 primarily regulated the expression of FAO-related genes, such as PPARα, ACOX-1, CPT-1α, rather than genes related to lipogenesis, fatty acid uptake, and lipid transport with or without TGF-β treatment in HK2 cells (Figs. 4D–F, S4C, D). The role of STAT6 in regulating lipid metabolism pathway was confirmed in primary renal tubular cells isolated from Stat6 WT and cKO mice. qPCR analysis showed that with deletion of STAT6, genes related to fibrotic related proteins were downregulated while genes related to FAO were upregulated (Figs. 4F, S4E, F). The role of STAT6 in regulating FAO was further confirmed by western blot in HK2 cells transfected with STAT6 siRNA or expression vector (Fig. 4G). All the data suggested that STAT6 in tubular cells was involved in kidney tubulointerstitial fibrosis partly by regulating FAO.

### STAT6 regulates FAO and fibrotic related protein expression by targeting PPARα in tubular cells

**Fig. 5:**
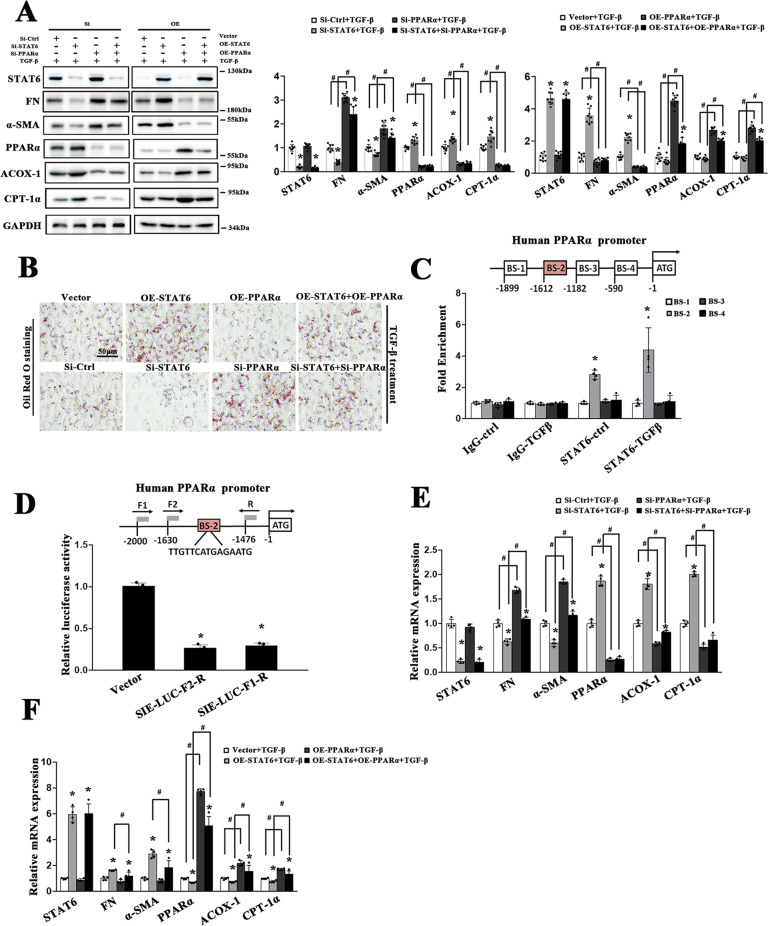
STAT6 regulates FAO and fibrotic related protein expression by targeting PPARα in tubular cells. HK2 cells were transfected with siRNA or plasmid for STAT6 or PPARα. After 24 h incubation, cells were treated with TGF-β (5 ng/ml) for another 24 h. **A** Cell lysates were harvested and subjected to immunoblot analyses with the indicated antibodies. Quantification of relative protein expression was determined. Results are expressed as the mean ± SD (*n* = 4 *p < 0.05, SiCtrl/Vector *vs*. siSTAT6/STAT6 transfection; #*p* < 0.05, SiCtrl/Vector *vs*. SiPPARα/PPARα transfection). **B** Representative micrographs showing the Oil Red O staining of indicated treatments in HK2 cells. **C** Identification of four SIEs in the promoter of PPARα. The potential site of STAT6 binding to PPARα promoter with detected by ChIP assay in HK2 cells with or without TGF-β (5 ng/ml) 24 h treatment. Results are expressed as the mean ± SD (*n* = 4 *p < 0.05, IgG *vs*. STAT6 immunoprecipitation). **D** The different human PPARα promoter constructs were cloned upstream of a luciferase reporter gene. HK2 cells were either transfected with empty vector or these constructs along with Renilla luciferase reporter for 24 h and followed by another 24 h TGF-β treatment, dual-luciferase activities were measured. The experiment was repeated three times, each with triplicate samples. Data are expressed as mean ± SD (*n* = 3, *p < 0.05, Vector *vs*. PPARα promoter constructs). **E**, **F** HK2 cells were transfected with indicated siRNA and plasmid for 24 h in serum-free medium, followed by TGF-β (5 ng/ml) 24 h and harvested for qRT-PCR analysis of the indicated genes. Results are expressed as the mean ± SD (*n* = 4 *p < 0.05, SiCtrl/Vector *vs*. siSTAT6/STAT6 transfection; ^#^*p* < 0.05, SiCtrl/Vector *vs*. SiPPARα/PPARα transfection).

PPI graph in Fig. S1Q has shown that PPARα, one of the major regulators in lipid metabolism, may serve as the linker between STAT6 and lipid metabolism. The potential involvement of PPARα was next explored. HK2 cells were transfected with siRNA or plasmids as indicated. As shown in Fig. 5A, B, downregulating STAT6 consistently activated PPARα and its target FAO genes with reduced lipid accumulation, accompanying reduction in fibrotic related genes expression, and the effects were inhibited by PPARα siRNA cotransfection. While overexpressed STAT6 and PPARα yielded the opposite results. These results indicated that STAT6 mediated the fibrotic genes expression partly through inhibiting PPARα regulated lipid metabolism. To unveil the detailed regulation of STAT6 on PPARα, we obtained the binding motif of STAT6 from JASPAR and screened four potential binding sequences for a ChIP experiment, as shown in Fig. 5C, Binding site-2 (BS-2) fragment (from −1612bp to −1598) was responsible for the STAT6-mediated PPARα promoter activity. Furthermore, the luciferase reporter gene assay data showed that HK2 cells transfected with the vectors containing the indicated fragments of PPARα promoter region along with Renilla luciferase reporter driven the reduction of luciferase activity (Fig. 5D). Consistently, cells with PPARα and/or STAT6 siRNA or plasmid transfection showed that STAT6-regulated FAO and fibrotic related genes through PPARα (Figs. 5E, F, S5).

### AS1517499 improves aberrant lipid metabolism and renal fibrosis after UUO

**Fig. 6:**
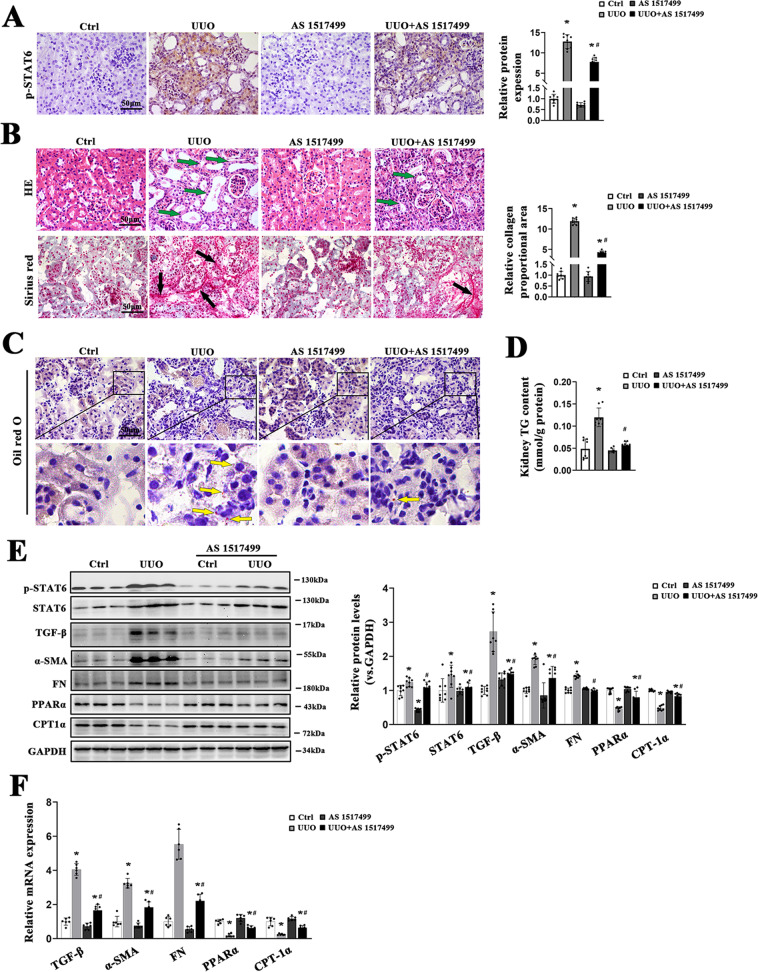
AS1517499 attenuates aberrant lipid metabolism and tubulointerstitial fibrosis in UUO mice. **A** Representative micrographs for p-STAT6 staining in the kidney from the indicated groups, and quantification of relative protein expression in the kidneys from the indicated groups. **B** Representative Sirius red staining and H&E staining in the kidney from the indicated groups, and quantification of relative collagen proportion in the kidneys from the indicated groups. **C** Representative Oil O Red staining in the kidney from the indicated groups. **D** TG content was determined in the kidneys from the indicated groups. **E** Kidney tissue lysates from each group were subjected to immunoblot analyses with the indicated antibodies. Representative blots of three independent samples in each group were shown and quantification of relative protein expression was determined. **F** The mRNA levels of genes related to lipid metabolism and renal fibrosis in the kidneys from the indicated groups. Results are expressed as the mean ± SD (*n* = 6-8, *p < 0.05, UUO *vs*. Ctrl, ^#^*p* < 0.05, UUO *vs*. UUO + AS1517499).

Then, we sought to explore whether pharmacological inhibition of STAT6 could alleviate the aberrant lipid metabolism and renal fibrosis caused by UUO. Mice with UUO received the STAT6 inhibitor AS1517499, a STAT6 selective inhibitor. Immunohistochemical staining revealed UUO-caused STAT6 activation was remarkably suppressed with AS1517499 treatment (Fig. 6A); H&E staining showed that the histological injury caused by UUO was attenuated by AS1517499, Sirius red staining exhibited the alleviated collagen deposition by AS1517499 (Fig. 6B). Besides, lipid accumulation was diminished after AS1517499 treatment as shown by Oil Red O staining and TG content in the kidney (Fig. 6C, D). Next, consistent with the staining results, immunoblot analyses indicated the expression of p-STAT6 was decreased in the kidney of mice with AS1517499 treatment, and the deactivation of STAT6 pathway further suppresses the expression of fibrosis-related protein and induce the expression of FAO-related protein in UUO kidney (Fig. 6E). Real-time-PCR assay showed that AS1517499 mainly induced the FAO in the kidney with or without UUO performance, and further alleviate renal fibrosis after UUO (Fig. 6F). These data suggested that AS1517499 attenuates UUO-induced renal fibrosis through STAT6 inhibition and FAO activation.

## Discussion

Several studies have indicated that alterations in metabolic function play an important role in the initiation and progression of renal fibrosis [6, 7, 20]. The results of the present study revealed that STAT6 inhibition promotes a shift to FAO for energy utilization by inducing PPARα activation in tubular cells and attenuates kidney interstitial fibrosis following UUO.

The role of STAT6 in kidney disease has not been clearly investigated. Previous reports indicated that STAT6-regulated cell differentiation participates in obstructive nephropathy, cystic disease, and ischemia–reperfusion injury [21–23]. In our previous study, we showed that STAT6 modulates TGF-β expression in tubular cells and causes subsequent increases in partial EMT and ECM protein expression after UUO [18]. However, the Stat6 KO mice used in this work were not tubular cell-specific KOs, which may affect the reliability of the results; in addition, the detailed mechanisms were not explored. Herein, we completely investigated the role of STAT6 in tubular cells in regulating kidney fibrosis. First, we generated tubular-specific-Stat6 gene KO mice. The proximal tubule is the primary target of various injury factors induced by kidney injury and the most important site of energy metabolism [5, 24, 25]. The γGT^Cre^ transgenic strain was consequently selected for further experiments. The tubular-specific-Stat6 KO mice displayed no obvious phenotypic and lipid metabolic changes compared with the wild-type (WT) controls within 2 months of birth, thus suggesting that STAT6 is dispensable for cell metabolic functions under normal physiological conditions. We then demonstrated that STAT6 inhibition in tubular cells could suppress kidney fibrosis. First, p-STAT6 was activated in tubular cells in multiple mouse models. Second, ECM deposition and histological changes revealed that ablation of tubular epithelium STAT6 alleviates kidney fibrosis. Third, ablation of STAT6 in primary STAT6 WT and KO tubular cells inhibited ECM protein expression in vitro; the same results were obtained in vivo by STAT6 deficiency. In addition to ablation of STAT6 signaling by genetic method, we treated mice with AS1517499, a potent and selective STAT6 inhibitor to pharmacologically block STAT6 signaling [26, 27]. p-STAT6 expression was significantly downregulated in tubular cells with AS1517499 treatment after UUO. We also observed the protective effects of AS1517499 against renal fibrosis by using H&E, Sirius red staining, western blot, and qPCR for α-SMA, FN. Thus, STAT6 could potentially serve as a therapeutic target for fibrotic renal disease.

The mechanism by which STAT6 inhibition in tubular cells protects against kidney fibrosis was explored. RNA sequencing of the UUO model and subsequent PPI network analysis showed close interactions among STAT6, PPARα, and the latter’s FAO-related genes. Proximal tubular epithelial cells contain rich mitochondria and rely mostly on mitochondrial oxidative phosphorylation during ATP synthesis [7, 28]. The predominant source of energy for tubular cells is FAO. Impaired FAO has been reported in experimental and clinical CKD specimens [6, 7, 29–31]. By utilizing primary culture of STAT6 WT and KO tubular cells or the STAT6 vector and siRNA-transfected HK2 cells, suggesting that PPARα, FAO-related genes, ATP synthesis, and TG content could be reversely regulated by STAT6. We verified these findings through in vivo experiments by either genetic ablation or pharmacologic blockade of STAT6. Thus far, the role of STAT6 in facilitating lipid metabolism has focused primarily on PPARγ and its targeted lipid synthesis [32, 33], Roberto R et.al. showed that blockade of STAT6 enhances PPARα signals [19]. In our study, we further identified the STAT6 binding site in the PPARα promoter in HK2 cells. Lipid synthesis related genes were not regulated by STAT6 in tubular cells, suggesting that STAT6 may play different roles in lipid metabolism in various cells and experimental conditions. Lipotoxicity has been proposed to be a trigger for renal fibrosis. Overexpression of CD36 in tubular epithelial cells in mice facilitates lipid accumulation, but this process, in itself, is insufficient to achieve spontaneous renal fibrogenesis [34]. Thus, besides lipid accumulation, defects in mitochondrial β-oxidation may play a major role in regulating kidney fibrosis. The deficiency of metabolic transcription factors in tubular cells has been associated with the progression of renal fibrosis [7, 12]. Taking these results together, novel therapeutic treatment strategies aiming to improve metabolic alterations in tubular cells appear to be promising.

In summary, our findings uncovered the crucial role of STAT6 in regulating PPARα-mediated FAO in tubular cells and kidney interstitial fibrosis. Thus, inhibition of STAT6 in tubular cells may be a therapeutic target for preventing CKD progression.

## Materials and methods

### Animals and treatments

Six to eight weeks old male C57BL/6 mice were purchased from SLAC Laboratory Animal Co. Ltd. Stat6^flox/flox^ mice were from Cyagen Biosciences. All mice were housed with a standard 12 h light/dark cycle, climate-controlled and pathogen-free facility. Mice handling procedures in this work followed the Guide for the Care and Use of Laboratory Animals and the study protocols were approved by Soochow University Institutional Animal Care and Use Committee. To generate tubular-specific-Stat6, Stat6^flox/flox^ mice was crossed with γGT^Cre^ mice (Cyagen Biosciences). 8-week-old gender-matched wildtype and knockout mice from the same litter were selected randomly to indicated groups based on genotypes. Genotyping analyses were performed by PCR with genomic DNA isolated from mouse tails. Primers for genotyping were in the supplementary primer list. UUO performance was conducted as previously described [35]. HFD models were established by feeding a high-fat diet (60Kcal% High-fat Diets, D12492, Research Diets) continuously for 25w. I/R operation was performed using a previously established procedure [36], in brief, body temperature was maintained at 37 °C for the duration of ischemia, renal ischemia was induced using a microvascular clamp on the left renal pedicle for 30 min, and then the clamp was removed for reperfusion. Mice were euthanized at day 21 post-operation. Aristolochic acid I sodium salt (A9451, Sigma-Aldrich) was single administered intraperitoneally at a dose of 20 mg/kg, the same volume of saline was administered in the vehicle control group, and the mice were sacrificed after 14 days. Mice were divided into ten groups: (i) Ctrl (corresponds to UUO) with sham operation; (ii) Ctrl (corresponds to HFD) fed with normal standard permitted food (D12450J); (iii) Ctrl (corresponds to I/R) with sham operation; (iv) Ctrl (corresponds to AAN) with an injection of saline only; (v) UUO (sacrificed 7 days after the performance); (vi) HFD; (vii) AS1517499 (Selleck S8685; administered intraperitoneally every another day till the mice harvested, 10 mg/kg dissolved in 20% DMSO and 80% normal saline); (viii) I/R (ix) AAN; (x) AS1517499 + UUO. All the animals were sacrificed. Tissues and Serum were harvested and stored at −80 °C.

### Hematoxylin and eosin (H&E), immunohistochemical (IHC), Oil Red O, and Sirius red staining

Tissue sections were baked and deparaffinized. H&E staining was performed for pathological analysis. IHC staining was performed with EnVision+System—HRP kit (Dako) based on the manufacturer’s instruction. Oil Red O staining of cells and frozen tissue sections were performed using the kit from Solarbio according to the manufacture’s instructions. Sirius red staining was performed using Sirius red/Fast Green Collagen Staining Kit (Chondrex) according to the manufacture’s instructions. Images were collected and analyzed with a fluorescence microscope (Leica DM 2500).

### Cell culture and treatment

Human proximal renal tubular cell line HK2 was purchased from the Cell Bank of the Chinese Academy of Sciences. Cells were cultured in Dulbecco’s Modified Eagle’s Medium (DMEM, Corning) containing 10% FBS (Hyclone) in a 5% CO2 incubator at 37 °C. The identities were confirmed and cultured as recommended by the suppliers. Renal primary tubular epithelial cells were obtained from wild‐type and tubular-specific-STAT6 knockout mice. As described previously [37], kidneys were surgically removed from anesthetized mice (2 weeks old), and the renal cortices were sliced and digested with collagenase II (0.5 mg/ml) in Krebs–Henseleit–HEPES(KHS) buffer. The purified cells were grown in DMEM/F12 supplemented with 10% FBS, 2ul/ml insulin-transferrin‑selenium, 36 ng/ml Hydrocortisone, 100IU/ml penicillin, and 100 μg/ml streptomycin. To induce the fibrotic proteins expression and lipid accumulation, TGF-β (5 ng/ml) was added to the medium for 24 h after free serum culture.

### Metabolic and kidney function assays

Levels of TG, total cholesterol (TC), low-density lipoprotein cholesterol (LDL), and high-density lipoprotein cholesterol (HDL) were measured using corresponding commercial determination kits (all from Nanjing Jiancheng; TG: A110-1-1; TC: A111-1-1; LDL: A113-1-1; HDL: A112-1-1) with kidney tissue or serum samples. Cellular TG levels were measured using a commercial TG Quantification Colorimetric Kit (Biovision; K622) according to the manufacturer’s protocol.

### Protein analyses

For immunoblot analyses, cells and tissues were lysed in sample buffer (62.5 mM Tris-HCl [pH 6.9], 3% SDS, 10% glycerol, 5% Beta-mercaptoethanol, and 0.1% bromophenol blue). The lysates were sonicated and boiled for 10 min, then denatured lysates were electrophoresed through an SDS-polyacrylamide gel and subjected to immunoblot analysis. The following antibodies were used: STAT6 (sc-374021), p-STAT6 (sc-136019), Arg-1 (sc-166920), TGF-β (sc-146), α-SMA (sc-53142), FN (sc-18827), H3 (sc-517576), GAPDH (sc-32233) from Santa Cruz Biotechnology. For immunoprecipitation and ubiquitination analysis, cells were harvested with RIPA buffer (Thermo) and incubated with indicated antibodies (1 μg) together and protein A agarose beads (Invitrogen) at 4 °C overnight. Immunoprecipitated complexes were subjected to immunoblot with the indicated antibodies. Relative immunoblot bands were compared using the prestained protein marker (Vazyme Biotech Co.,Ltd, MP102) and Thermo Scientific PageRuler Prestained Protein Ladder.

### Nuclear protein extraction

The nuclear protein was isolated from indicated tissue using the Nuclear/Cytosolic Extraction Kit (CWBiotech, China, CW0199S) in accordance with the manufacturer’s instruction. Briefly, tissues were treated with Nc-Buffer A plus B (with 1% protease inhibitor). Followed by homogenization and centrifugation, the cytoplasmic protein was collected. And nuclear protein was extracted by Nc-Buffer C (with 1% protease inhibitor). Nuclear protein was subjected to western blot analysis as indicated with the prestained protein marker.

### Measurements of OCR

Oxygen consumption rates (OCR) were measured using the Seahorse XF24 Extracellular Flux analyzer (Agilent Technologies, Inc). HK2 cells were plated at 1 × 10^5^ cells/well onto an XF24 cell culture microplate (Agilent Technologies, Inc). Cells were sequentially exposed to oligomycin (1 μM), carbonyl cyanide p-(trifluoromethoxy) phenylhydrazone (FCCP; 150 μM), and Antimycin (1 µM)/ antimycin A(1 µM) combination. The data were plotted and analyzed automatically by Seahorse XF24 software.

### Data processing

The high throughout RNA sequence series matrix of GSE145053 [38] was obtained from Gene Expression Omnibus (ncbi.nlm.nih.gov/geo/). The protein-coding genes of GSE145053 were processed by DESeq2 [39] R package to identify the log fold change, *p*-value and adjusted *p*-value of each gene expression, in which genes that meet |log_2_FC | >1.5 and adjusted *p* < 0.05 are considered to be differentially expressed. A volcano plot and a heatmap created by R were graphed to show the overall condition of the data. Similarly, the normalized microarray series matrix was processed by limma [40] R package to calculate the log fold change, *p*-value and adjusted *p*-value of gene expression for further analysis, and genes that meet | log_2_FC | >1 and *p* < 0.05 are considered differentially expressed. A heatmap created by R is graphed to show the overall condition of the data.

### Functional enrichment analysis

The upregulated and downregulated genes in GSE118337 were respectively used for biological process functional enrichment on Metascape [41], the result of which was used to create bar plots showing functions related to the present investigation by R.

### Protein–protein interaction (PPI) analysis

Differentially expressed protein-coding genes from GSE145053 were used to create a PPI network on String (string-db.org/) [42], then visualized by Cytoscape 3.8.2 [43]. MCODE [44] was employed to identify highly interconnected regions in the whole network, each of which was examined on Metascape to identify its function. Regions related to the present investigation were highlighted, the genes in which, together with STAT6, were used to create a new network on String and visualized by Cytoscape to show the connection between them.

### RNA extraction and real-time RT-PCR

RNA isolated from cells and kidney tissues were obtained using TRIzol reagent purchased from CWBIO. cDNA was acquired with equal amounts of RNA using a HiFiScript cDNA synthesis kit according to the manufacturer’s instructions (CWBIO). ABI 7500 (Applied Biosystems) was used to evaluate RNA expression using an UltraSYBR Mixture qPCR kit(CWBIO) with 96-well PCR plates (Nest, 402601) as previously described [18]. Primer sequences are listed in the supplementary.

### Construction of recombinant DNA molecules

Deletion fragments of the human PPARα promoter sequence were amplified by PCR from genomic DNA extracted from HK2 cells and cloned into pGL4.22 (Promega) with the primers list in the supplementary. All the sequences were confirmed by direct nucleotide sequencing

### Transfection of siRNA and cDNA

Cells were transfected with the indicated small interfering RNA(siRNA) by HiperFect Transfection Reagent(Qiagen) according to the manufacturer’s instructions. Non-target siRNA, STAT6 siRNA, and PPARα siRNA were purchased from GenePharma and the target sequences are as follows: STAT6-gcaggaagaactcaagtttaa; PPARα-gcccgttatctgaagagtttt. Briefly, 20 pmol of siRNA and 12 μl transfection reagent were mixed in 100 μl Optimedium (Invitrogen). After 10 min room temperature incubation, mixtures were added into the cells. Cells were performed for the following indicated studies after 24 h incubation (37 °C; 5% CO_2_).

### Luciferase reporter gene assay

HK2 cells were co-transfected with the indicated deletion fragments of PPARα promoter luciferase constructs along with thymidine kinase(TK)-Renilla luciferase (internal control, Promega). After 24 h incubation, cells were left untreated or treated with TGF-β (5 ng/ml) for another 24 h. Luciferase activities were measured with the dual-luciferase reporter assay system (Beyotime).

### Chromatin immunoprecipitation (ChIP) assays

HK2 cells were stimulated with TGF-β (5 ng/ml) for 24 h prior to crosslinking for 10 min with 1% formaldehyde. Antibody recognizing STAT6 (sc-374021) and normal mouse IgG (negative control; sc-2025) were purchased from Santa Cruz. Magna Chip protein A Magnetic Beads (16-661) was purchased from Millipore. PPARα promoter PCR was performed with the specific primers flanking the STAT6 potential binding sites, and the sequences were showed in the supplementary primer list.

### Statistical analysis

The investigators were blinded to group allocation. Data were present as mean ± SD; Statistical analyses were performed using Prism 7 software. Unpaired Student’s *t-tests* were applied for comparison between two groups. For multiple comparison analyses, one-way ANOVA with *Bonferroni’s* correction were performed, variance was similar between groups. The differences with *p < 0.05 were considered statistically significant.

## Supplementary information

SUPPLEMENTAL MATERIAL

SUPPLEMENTAL Figure1

SUPPLEMENTAL Figure2

SUPPLEMENTAL Figure3

SUPPLEMENTAL Figure4

SUPPLEMENTAL Figure5

Checklist

Author Contribution Statement

## Data Availability

All data generated or analyzed during this study are included in this published article and its supplementary information files. The datasets used and analyzed during the current study are available from the corresponding author on reasonable request.

## References

[1] Zhang L, Zhao MH, Zuo L, Wang Y, Yu F, Zhang H (2011). China kidney disease network (CK-NET) 2016 annual data report. Kidney Int Suppl.

[2] Woo KT, Choong HL, Wong KS, Tan HB, Chan CM (2012). The contribution of chronic kidney disease to the global burden of major noncommunicable diseases. Kidney Int.

[3] Djudjaj S, Boor P (2019). Cellular and molecular mechanisms of kidney fibrosis. Mol Asp Med.

[4] LeBleu VS, Taduri G, O’Connell J, Teng Y, Cooke VG, Woda C (2013). Origin and function of myofibroblasts in kidney fibrosis. Nat Med.

[5] Gewin LS (2018). Renal fibrosis: primacy of the proximal tubule. Matrix Biol.

[6] Simon N, Hertig A (2015). Alteration of fatty acid oxidation in tubular epithelial cells: from acute kidney injury to renal fibrogenesis. Front Med (Lausanne).

[7] Kang HM, Ahn SH, Choi P, Ko YA, Han SH, Chinga F (2015). Defective fatty acid oxidation in renal tubular epithelial cells has a key role in kidney fibrosis development. Nat Med.

[8] Bougarne N, Weyers B, Desmet SJ, Deckers J, Ray DW, Staels B (2018). Molecular actions of PPARalpha in lipid metabolism and inflammation. Endocr Rev.

[9] Auboeuf D, Rieusset J, Fajas L, Vallier P, Frering V, Riou JP (1997). Tissue distribution and quantification of the expression of mRNAs of peroxisome proliferator-activated receptors and liver X receptor-alpha in humans: no alteration in adipose tissue of obese and NIDDM patients. Diabetes.

[10] Nguyen TTT, Shang E, Shu C, Kim S, Mela A, Humala N (2021). Aurora kinase A inhibition reverses the Warburg effect and elicits unique metabolic vulnerabilities in glioblastoma. Nat Commun.

[11] Chau BN, Xin C, Hartner J, Ren S, Castano AP, Linn G (2012). MicroRNA-21 promotes fibrosis of the kidney by silencing metabolic pathways. Sci Transl Med.

[12] Chung KW, Lee EK, Lee MK, Oh GT, Yu BP, Chung HY (2018). Impairment of PPARalpha and the fatty acid oxidation pathway aggravates renal fibrosis during aging. J Am Soc Nephrol.

[13] Fu C, Jiang L, Hao S, Liu Z, Ding S, Zhang W (2019). Activation of the IL-4/STAT6 signaling pathway promotes lung cancer progression by increasing M2 myeloid cells. Front Immunol.

[14] Binnemars-Postma K, Bansal R, Storm G, Prakash J (2018). Targeting the Stat6 pathway in tumor-associated macrophages reduces tumor growth and metastatic niche formation in breast cancer. FASEB J.

[15] Wang X, Ji Y, Feng P, Liu R, Li G, Zheng J (2021). The m6A reader IGF2BP2 regulates macrophage phenotypic activation and inflammatory diseases by stabilizing TSC1 and PPARgamma. Adv Sci (Weinh).

[16] Gu F, Wang C, Wei F, Wang Y, Zhu Q, Ding L (2018). STAT6 degradation and ubiquitylated TRIML2 are essential for activation of human oncogenic herpesvirus. PLoS Pathog.

[17] Cai W, Dai X, Chen J, Zhao J, Xu M, Zhang L, et al. STAT6/Arg1 promotes microglia/macrophage efferocytosis and inflammation resolution in stroke mice. JCI Insight. 2019;4:e131355. 10.1172/jci.insight.131355 PMC6824303 31619589

[18] Li J, Yang Y, Wei S, Chen L, Xue L, Tian H (2020). Bixin protects against kidney interstitial fibrosis through promoting STAT6 degradation. Front Cell Dev Biol.

[19] Ricardo-Gonzalez RR, Red Eagle A, Odegaard JI, Jouihan H, Morel CR, Heredia JE (2010). IL-4/STAT6 immune axis regulates peripheral nutrient metabolism and insulin sensitivity. Proc Natl Acad Sci USA.

[20] Breyer MD, Susztak K (2016). The next generation of therapeutics for chronic kidney disease. Nat Rev Drug Disco.

[21] Yan J, Zhang Z, Yang J, Mitch WE, Wang Y (2015). JAK3/STAT6 stimulates bone marrow-derived fibroblast activation in renal fibrosis. J Am Soc Nephrol.

[22] Zhang MZ, Wang X, Wang Y, Niu A, Wang S, Zou C (2017). IL-4/IL-13-mediated polarization of renal macrophages/dendritic cells to an M2a phenotype is essential for recovery from acute kidney injury. Kidney Int.

[23] Olsan EE, Mukherjee S, Wulkersdorfer B, Shillingford JM, Giovannone AJ, Todorov G (2011). Signal transducer and activator of transcription-6 (STAT6) inhibition suppresses renal cyst growth in polycystic kidney disease. Proc Natl Acad Sci USA.

[24] Liu BC, Tang TT, Lv LL (2019). How tubular epithelial cell injury contributes to renal fibrosis. Adv Exp Med Biol.

[25] Liu BC, Tang TT, Lv LL, Lan HY (2018). Renal tubule injury: a driving force toward chronic kidney disease. Kidney Int.

[26] Chen J, Li Y, Lai F, Wang Y, Sutter K, Dittmer U (2021). Functional comparison of interferon-alpha subtypes reveals potent hepatitis B virus suppression by a concerted action of interferon-alpha and interferon-gamma signaling. Hepatology.

[27] Li C, Qiu S, Jin K, Zheng X, Zhou X, Jin D (2021). Tumor-derived microparticles promote the progression of triple-negative breast cancer via PD-L1-associated immune suppression. Cancer Lett.

[28] Meyer C, Nadkarni V, Stumvoll M, Gerich J (1997). Human kidney free fatty acid and glucose uptake: evidence for a renal glucose-fatty acid cycle. Am J Physiol.

[29] Li S, Mariappan N, Megyesi J, Shank B, Kannan K, Theus S (2013). Proximal tubule PPARalpha attenuates renal fibrosis and inflammation caused by unilateral ureteral obstruction. Am J Physiol Ren Physiol.

[30] Szeto HH (2017). Pharmacologic approaches to improve mitochondrial function in AKI and CKD. J Am Soc Nephrol.

[31] Herman-Edelstein M, Scherzer P, Tobar A, Levi M, Gafter U (2014). Altered renal lipid metabolism and renal lipid accumulation in human diabetic nephropathy. J Lipid Res.

[32] Jun I, Kim BR, Park SY, Lee H, Kim J, Kim EK (2020). Interleukin-4 stimulates lipogenesis in meibocytes by activating the STAT6/PPARgamma signaling pathway. Ocul Surf.

[33] Sajic T, Hainard A, Scherl A, Wohlwend A, Negro F, Sanchez JC (2013). STAT6 promotes bi-directional modulation of PKM2 in liver and adipose inflammatory cells in rosiglitazone-treated mice. Sci Rep..

[34] Yang X, Okamura DM, Lu X, Chen Y, Moorhead J, Varghese Z (2017). CD36 in chronic kidney disease: novel insights and therapeutic opportunities. Nat Rev Nephrol.

[35] Li J, Ren J, Liu X, Jiang L, He W, Yuan W (2015). Rictor/mTORC2 signaling mediates TGFbeta1-induced fibroblast activation and kidney fibrosis. Kidney Int.

[36] Xu L, Sharkey D, Cantley LG (2019). Tubular GM-CSF promotes late MCP-1/CCR2-mediated fibrosis and inflammation after ischemia/reperfusion injury. J Am Soc Nephrol.

[37] Sun X, Wei W, Ren J, Liang Y, Wang M, Gui Y (2019). Inhibition of 4E-BP1 phosphorylation promotes tubular cell escaping from G2/M arrest and ameliorates kidney fibrosis. Cell Signal.

[38] Conway BR, O’Sullivan ED, Cairns C, O’Sullivan J, Simpson DJ, Salzano A (2020). Kidney single-cell atlas reveals myeloid heterogeneity in progression and regression of kidney disease. J Am Soc Nephrol.

[39] Love MI, Huber W, Anders S (2014). Moderated estimation of fold change and dispersion for RNA-seq data with DESeq2. Genome Biol.

[40] Ritchie ME, Phipson B, Wu D, Hu Y, Law CW, Shi W (2015). limma powers differential expression analyses for RNA-sequencing and microarray studies. Nucleic Acids Res.

[41] Zhou Y, Zhou B, Pache L, Chang M, Khodabakhshi AH, Tanaseichuk O (2019). Metascape provides a biologist-oriented resource for the analysis of systems-level datasets. Nat Commun.

[42] Szklarczyk D, Gable AL, Lyon D, Junge A, Wyder S, Huerta-Cepas J (2019). STRING v11: protein-protein association networks with increased coverage, supporting functional discovery in genome-wide experimental datasets. Nucleic Acids Res.

[43] Shannon P, Markiel A, Ozier O, Baliga NS, Wang JT, Ramage D (2003). Cytoscape: a software environment for integrated models of biomolecular interaction networks. Genome Res.

[44] Bader GD, Hogue CW (2003). An automated method for finding molecular complexes in large protein interaction networks. BMC Bioinforma.

