# Supplementary material for: STAT6 contributes to renal fibrosis by modulating PPARα-mediated tubular fatty acid oxidation
Source: Cell Death Dis. 2022 Jan 19;13(1):66. doi: 10.1038/s41419-022-04515-3 (PMC8770798; doi:10.1038/s41419-022-04515-3)
Supplement: Supplementary file 1 — SUPPLEMENTAL MATERIAL [file 41419_2022_4515_MOESM1_ESM.docx]

**Supplementary Information**

**Contents**

Supplementary primer list

Supplementary Figure S1

Supplementary Figure S2

Supplementary Figure S3

Supplementary Figure S4

Supplementary Figure S5

**Supplementary figure legends**

**Figure S1. UUO-induced renal fibrotic gene and damped fatty acid oxidation.** (A) Schematic illustration of the UUO and HFD models. (B-F) Comparison of BW, KW, KW/BW, BUN, Serum lipid level between the UUO and sham-operated groups. (G-K) Comparison of BW, KW, KW/BW, BUN, Serum lipid level between the control and HFD groups. Results are expressed as means ±SD (**p*<0.05, Ctrl *vs.* HFD, n=8). (L) The volcano plot shows the overall condition of 13071 protein-coding genes, which is graphed with log2 fold change and -lg adjusted *p* value by R. The intercept lines are consistent with filter condition of differentially expressed genes. A few genes related to fibrosis and fatty acid metabolism are labelled. (M) The heatmap shows the expression of top 50 upregulated genes and top 50 downregulated genes in 4 samples of UUO group by z-score, in comparison with control group. (N) The biological process functional enrichment result of the fibrosis related protein-coding genes in S1Q. (O) The biological process functional enrichment result of the fatty acid related protein-coding genes in S1Q. (P) The PPI network shows the relationship of the proteins of the top 10 clusters created by MCODE in 1682 differentially expressed genes, with fibrosis related proteins and fatty acid metabolism related proteins highlighted. (Q) The PPI network shows the interaction of fibrosis related proteins and fatty acid metabolism related proteins with STAT6.

**Figure S2. STAT6 is activated in the kidneys of I/R and AAN mice.** (A) Representative micrographs for IHC staining of p-STAT6 in kidney section and quantification are performed. (B)The protein expression of p-STAT6, Arg-1, TGF-β, α-SMA, FN in the indicated groups were determined by immunoblot analyses with the quantification on the right panel. (C) Representative micrographs for Sirius Red and relative collagen proportion was quantified. (D) Representative micrographs for H&E and Oil Red O staining in kidney sections from indicated group. (E) Kidney TG content were measured in the indicated groups Results are expressed as the mean ± SD (n=8, *p<0.05, Ctrl vs. Treatments).

**Figure S3. The effects of STAT6 deficiency in tubular cells at basal level.** (A) Immunoblot analysis showed the abundance of STAT6, Arg-1, α-SMA in the kidney lysates from above WT and cKO mice and quantification of relative protein expression was determined. (B-D) Comparison of BW, KW, KW/BW between above WT and cKO mice at 2 months after birth (n=8, data are presented as mean ± SD. **p*<0.05, WT *vs.* KO).

**Figure S4. STAT6 inhibited PPARα/FAO at the basal level in renal tubular cells.** HK2 cells were transfected with siRNA or plasmid for STAT6 inhibition or overexpression. After 24h serum free medium culture. (A) The mRNA levels of STAT6 in HK2 cells. (N=4, data are presented as mean ± SD **p*<0.05, compared with those cells treated with scramble siRNA or vector plasmid.)(B) TG content was determined enzymatically. (N=4, data are presented as mean ± SD **p*<0.05, compared with those cells treated with scramble siRNA or vector plasmid.) (C-D) The mRNA levels of genes related to lipid metabolism in STAT6 inhibited or overexpressed HK2 cells were determined by qRT-PCR, data are presented as mean ± SD. (N=4 **p*<0.05, compared with those cells treated with scramble siRNA or vector plasmid.) (E) Primary renal tubular epithelial cells were isolated from Stat6 WT and cKO kidneys, and followed by TGF-β (5ng/ml) treatment for 24h. Cell lysates were harvested for qRT-PCR, the mRNA levels of genes related to lipid metabolism were measured data are presented as mean ± SD. N=4, **p*<0.05, WT *vs.* cKO primary cells; #*p*<0.05, Ctrl *vs*. TGF-β (F) The mRNA levels of genes related to FAO and fibrotic proteins expression in primary renal tubular epithelial cells isolated from Stat6 WT and cKO kidneys (n=4, Data are presented as mean ± SD; **p*<0.05, compared with WT Ctrl cells).

**Figure S5. STAT6 mediates lipid metabolism related genes.** HK2 cells were transfected with siRNA or plasmid for STAT6 or PPARα inhibition or overexpression. The mRNA levels of genes related to lipid metabolism were determined by qRT-PCR, data are presented as mean ± SD (n=4, **p*<0.05,siCtrl *vs.* siSTAT6; #*p*<0.05, siCtrl *vs.* siPPARα).

Supplementary primer list

| Human qPCR Primer list | | Sequences |
| --- | --- | --- |
| h‐SREBP-1c | Forward | TGCATTTTCTGACACGCTTC |
|  | Reverse | CCAAGCTGTACAGGCTCTCC |
| h-SREBP-2 | Forward | CCGCCTGTTCCGATGTACAC |
|  | Reverse | TGCACATTCAGCCAGGTTCA |
| h-FASN | Forward | GGAAGCTGCCAGAGTCGGAGAACT |
|  | Reverse | TGAGGGTCCATCGTGTGTGCCT |
| h-PPARα | Forward | ATGGTGGACACGGAAAGCC |
|  | Reverse | CGATGGATTGCGAAATCTCTTGG |
| h-ACOX-1 | Forward | CCAAGCTTTCCTGCTCAGTGTT |
|  | Reverse | CCCCCAGTCCCTTTTCTTCA |
| h-CPT-1α | Forward | TCGTCACCTCTTCTGCCTTT |
|  | Reverse | ACACACCATAGCCGTCATCA |
| h-CD36 | Forward | GGCTTAATGAGACTGGGACCA |
|  | Reverse | TCACCACACCAACACTGAGT |
| h-FATP1 | Forward | TGACGTGCTCTATGACTGCC |
|  | Reverse | ACTTGATGCAGTCGTCCCAG |
| h-APOA4 | Forward | GGTGACCTGCAGAAGAAGCT |
|  | Reverse | CCAGCTCCTTCCCAATCTCC |
| h-MTTP | Forward | ATACCTGCAGCCTGACAACC |
|  | Reverse | TTCTTCGCAGTCCTGAGGTG |
| h-FN | Forward | TGGCTGTCAGTCAAAGCAAG |
|  | Reverse | CTCGGCTTCCTCCATAACAA |
| h-STAT6 | Forward | CACCGAGGGAATGGCGCACCGTTTG |
|  | Reverse | AAACCAAACGGTGCGCCATTCCCTC |
| h-α-SMA | Forward | CAGCCAAGCACTGTCAGG |
|  | Reverse | CCAGAGCCATTGTCACACAC |
| h-Arginase1 | Forward | GGCTGGTCTGCTTGAGAAAC |
|  | Reverse | TTCCCACAGACCTTGGATTC |
| h-TGFβ | Forward | GTACCTGAACCCGTGTTGCT |
|  | Reverse | GTATCGCCAGGAATTGTTGC |
| h-GAPDH | Forward | CTGACTTCAACAGCGACACC |
|  | Reverse | TGCTGTAGCCAAATTCGTTGT |

| Mouse qPCR Primer list | | Sequences |
| --- | --- | --- |
| m‐α-SMA | Forward | GTCCCAGACATCAGGGAGTAA |
|  | Reverse | TCGGATACTTCAGCGTCAGGA |
| m‐FN | Forward | CATGAAGGGGGTCAGTCCTA |
|  | Reverse | TAGGTTTGCAGGTCCATTCC |
| m‐β-actin | Forward | AAGGCCAACCGTGAAAAGAT |
|  | Reverse | GTGGTACGACCAGAGGCATAC |
| m‐SREBP-1c | Forward | GGGCAAGTACACAGGAGGAC |
|  | Reverse | AGATCTCTGCCAGTGTTGCC |
| m-TGFβ | Forward | GACTCTCCACCTGCAAGACC |
|  | Reverse | GACTGGCGAGCCTTAGTTTG |
| m-STAT6 | Forward | GATGACTGTGGAAAGGGACCA |
|  | Reverse | GGATGGACTGTGGAGGATACC |
| m-Arginase1 | Forward | CGCCTTTCTCAAAAGGACAG |
|  | Reverse | TTTTTCCAGCAGACCAGCTT |
| m-SREBP-2 | Forward | CAGGCGACCAGGAAGAAGAG |
|  | Reverse | CGGAACTGCTGGAGAATGGT |
| m-FASN | Forward | GACCTCAGGCTGCAGTGAAT |
|  | Reverse | CACCTTCTTGAGAGCCTGCA |
| m-PPARα | Forward | AGTGCCCTGAACATCGAGTG |
|  | Reverse | TTGCAGCTCCGATCACACTT |
| m-ACOX-1 | Forward | GGGGAACATCATCACAGGGG |
|  | Reverse | ATCATAGCGGCCGAGAACAG |
| m-CPT-1α | Forward | TTGGACGAATCGGAACAGGG |
|  | Reverse | CCATGCAGCAGAGATTTGGC |
| m-CD36 | Forward | ATTGTACCTGGGAGTTGGCG |
|  | Reverse | CAGCCAGGACTGCACCAATA |
| m-FATP1 | Forward | CGCCGATGTGCTCTATGACT |
|  | Reverse | ACACAGTCATCCCAGAAGCG |
| m-APOA4 | Forward | GGAGCACCTGAAGCCCTATG |
|  | Reverse | CATCATCGAGGTGTGCAGGT |
| m-MTTP | Forward | AATGCGGGTCAACAGAGAGG |
|  | Reverse | CCCCGGACCAGATGAAGAAG |

| Chip Primer list | | Sequences |
| --- | --- | --- |
| BS-1 | Forward | CTTCGTGGCCAACAGAAAAT |
|  | Reverse | TCAAATGCTGTCAGCCAGTC |
| BS-2 | Forward | CCCACATAGCCTTTGTCCTT |
|  | Reverse | TCCGGGCTCAAAGACATTAC |
| BS-3 | Forward | GCAAGAGAAGGTGAGGTTGC |
|  | Reverse | CTCATTAGGCAGGGGAACTG |
| BS-4 | Forward | CCACCTGTTTCCTTGTCCTC |
|  | Reverse | TGCTACTCTGTGCGATGAGC |

| Primers for Luciferase reporter cloning  (PPARα promoter fragments) | | Sequences |
| --- | --- | --- |
| F1 | Forward | GGGGTACCGGGGGTGAGGGTGT |
| F2 | Forward | GGGGTACCCCCACATAGCCTTTGTCCTT |
| R | Reverse | CCGCTCGAGTCCGGGCTCAAAGACATTAC |

| Primers for genotyping | | Sequences |
| --- | --- | --- |
| Stat6 | Forward | TATCAAATGGCCTAGACTTGGTGG |
|  | Reverse | GCTTCTGAGACTCAAGTTATAGCA |
| Cre | Forward | GAACGCACTGATTTCGACCA |
|  | Reverse | GCTAACCAGCGTTTTCGTTC |
