# Supplementary figures and images for: STAT6 contributes to renal fibrosis by modulating PPARα-mediated tubular fatty acid oxidation
Source: Cell Death Dis. 2022 Jan 19;13(1):66. doi: 10.1038/s41419-022-04515-3 (PMC8770798; doi:10.1038/s41419-022-04515-3)

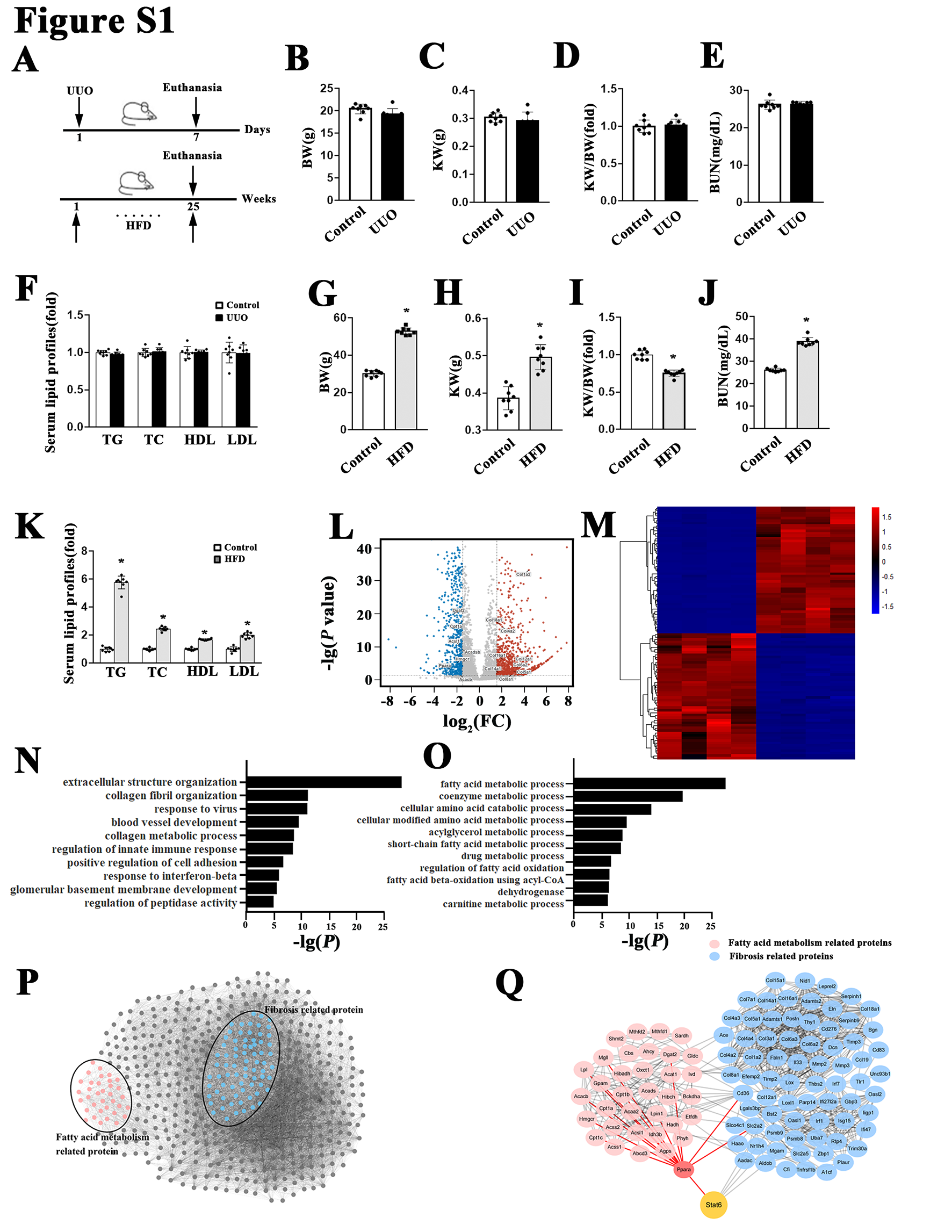

Supplement: Supplementary file 2 — SUPPLEMENTAL Figure1 [file 41419_2022_4515_MOESM2_ESM.tif]

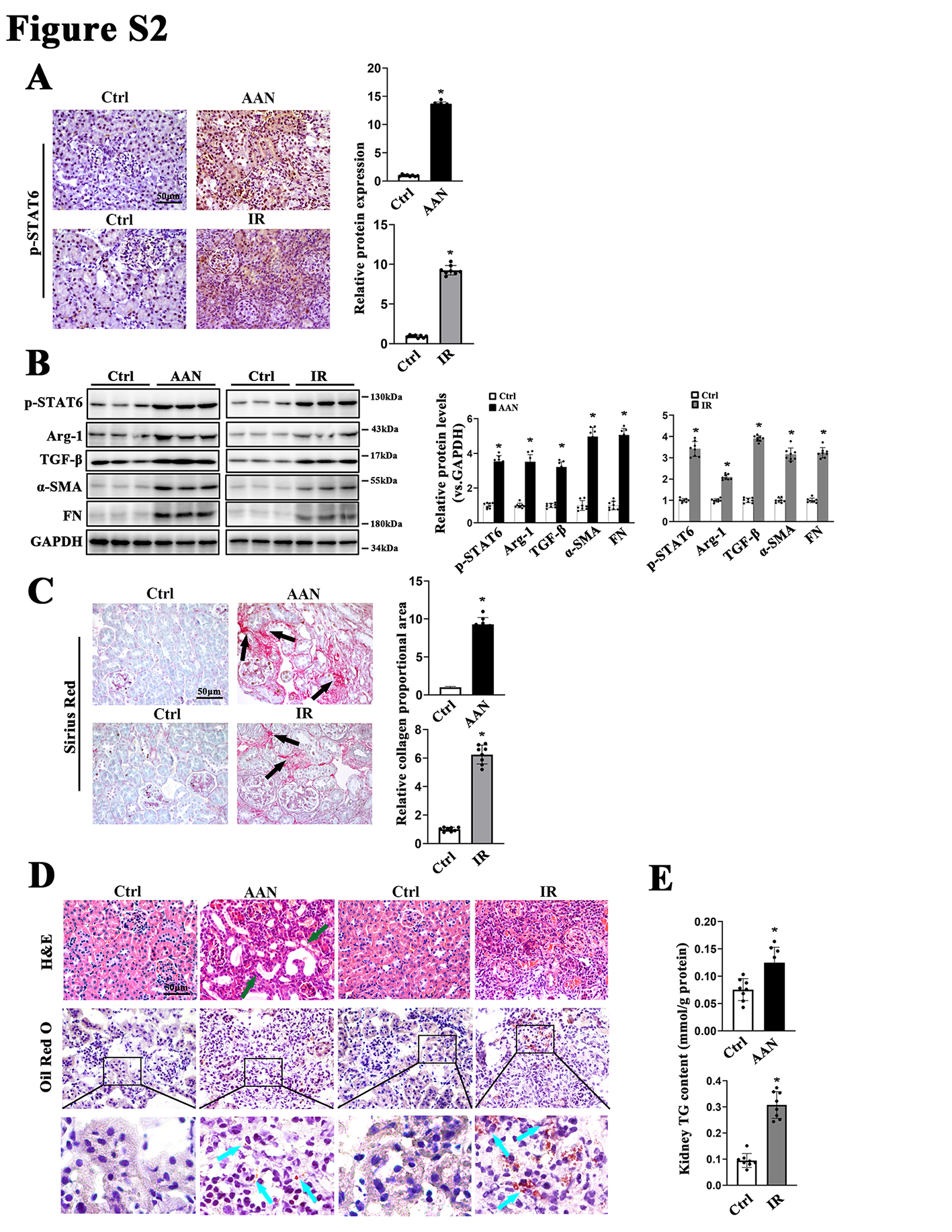

Supplement: Supplementary file 3 — SUPPLEMENTAL Figure2 [file 41419_2022_4515_MOESM3_ESM.tif]

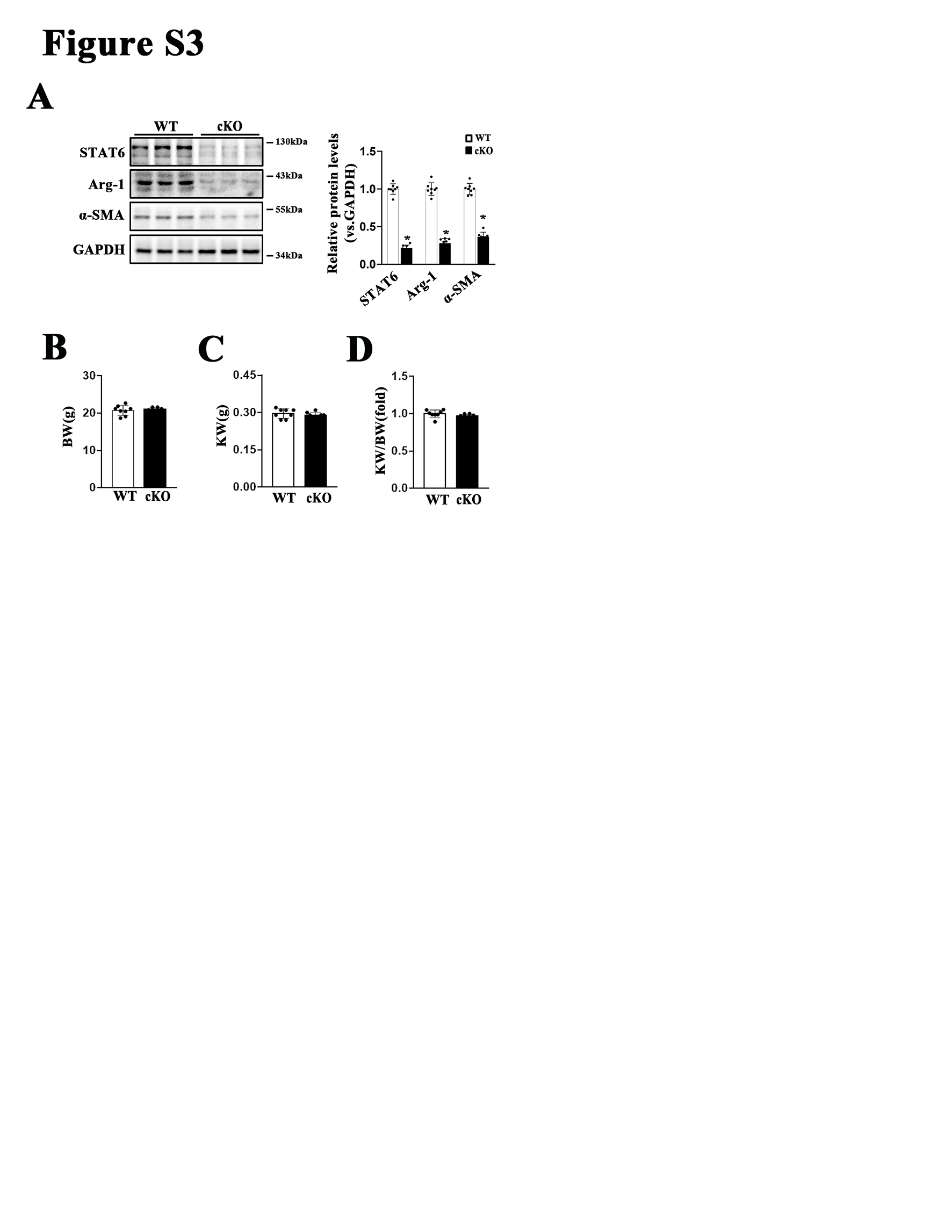

Supplement: Supplementary file 4 — SUPPLEMENTAL Figure3 [file 41419_2022_4515_MOESM4_ESM.tif]

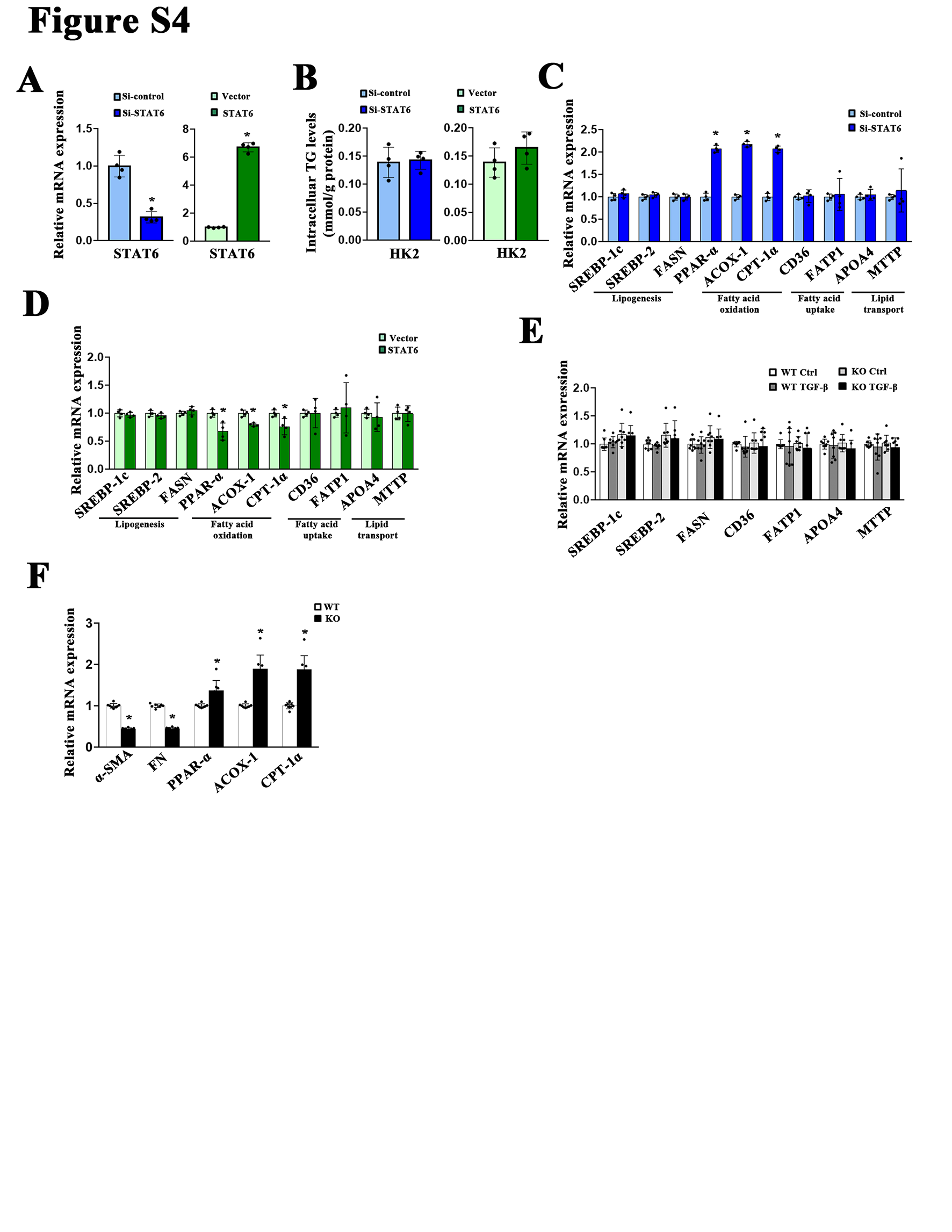

Supplement: Supplementary file 5 — SUPPLEMENTAL Figure4 [file 41419_2022_4515_MOESM5_ESM.tif]

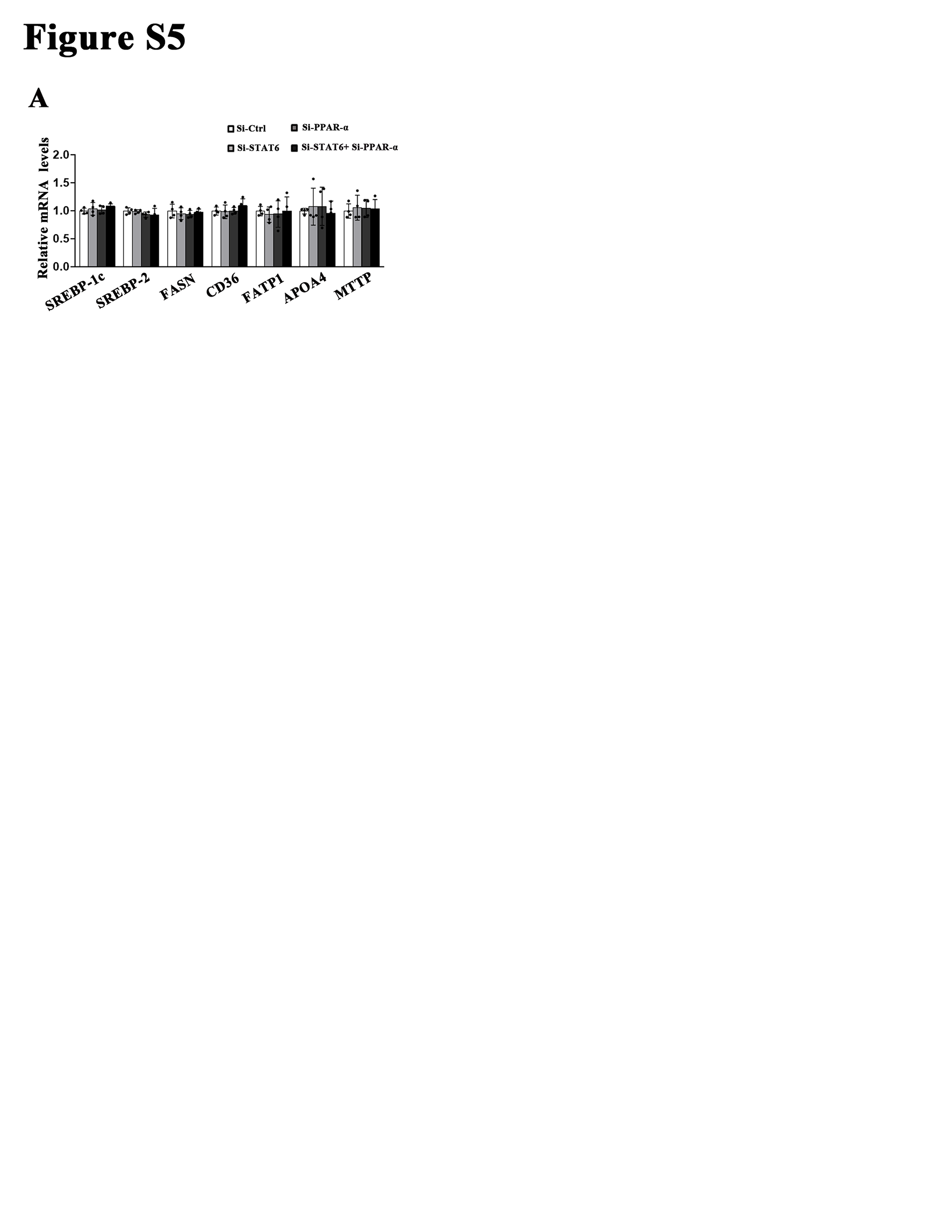

Supplement: Supplementary file 6 — SUPPLEMENTAL Figure5 [file 41419_2022_4515_MOESM6_ESM.tif]
