# Supplementary material for: STAT6 contributes to renal fibrosis by modulating PPARα-mediated tubular fatty acid oxidation
Source: Cell Death Dis. 2022 Jan 19;13(1):66. doi: 10.1038/s41419-022-04515-3 (PMC8770798; doi:10.1038/s41419-022-04515-3)
Supplement: Supplementary file 8 — Author Contribution Statement [file 41419_2022_4515_MOESM8_ESM.pdf]

DECLARATION OF CONTRIBUTIONS TO ARTICLE

ADMC

Manuscript Number:

Journal Name: *Cell Death & Differentiation* (the 'Journal')

Proposed Title of the Contribution:

STAT6 contributes to renal fibrosis by modulating PPARα-mediated tubular fatty acid oxidation (the 'Contribution)

Author(s):

Jianzhong Li , Youjing Yang , Shuhui Wei , Yujia Zhou , Wangjianfei Yu Qianmin Li , Lian Xue , Ling Zhou , Lei Shen , Guoyuan Lu , Ling Chen , Shasha Tao (the 'Authors')

For all *CDD* articles, each person named as an author in the published version must be able to show he or she has contributed substantially to the article.

Authorship credit should be based on 1) substantial contributions to conception and design, acquisition of data, or analysis and interpretation of data; 2) drafting the article or revising it critically for important intellectual content; and 3) final approval of the version to be published. Authors should meet conditions 1, 2 and 3.

Any person who cannot be shown to have made a substantial contribution to the article cannot be listed as an author in the final version. The name of any person who is deemed to have made a minor contribution can, however, appear in the Acknowledgments section of the article.

Please complete the table below to indicate the contributions of all named authors to the manuscript.

Author Full Name:	Specification of Contribution to the Manuscript:
Shasha Tao	Supervised the whole project, critically edited the manuscript text and final approved the version to be published.
Jianzhong Li	designed the experiments,conducted the experiments and co-wrote the manuscript.
Youjing Yang	Conducted the experiments and analyzed the data,co-wrote the manuscript..
Shuhui Wei	conducted the experiments, and co-wrote the manuscript
Lian Xue	designed the experiments
Yujia Zhou	Conducted the experiments and analyzed the data,co-wrote the manuscript..
Wangjianfei Yu	analyze the data
Qianmin Li	analyze the data
Ling Chen	analyze the data
Ling Zhou	conducted the experiments, and co-wrote the manuscript
Lei Shen	analyze the data.
Guoyuan Lu	analyze the data

Please complete the table below to indicate the contributions of all named authors to the figures.

Figure 1:

In Figure 1, Jianzhong Li and Youjing Yang generated the data ; Jianzhong Li and Ling Zhou generated the data of Figure S1 and Shasha Tao checked the data.

Figure 2:

In Figure 2, Jianzhong Li generated the data ; In Figure S2, Shuhui Wei and Youjing Yang generated all the data. Shasha Tao and Lian Xue assembled the Figure 2 & Figure S2.

Figure 3:

In Figure 3, Jianzhong Li and Youjing Yang generated the data In Figure S3, Shuhui Wei ,Qianmin Li, Ling Chen generated the data, In Figure 3S,Youjing Yang and Jianzhong Li generated the data . Shasha Tao analyzed all the data.

Figure 4:

In Figure 4 and Figure S4, Jianzhong Li, Youjing Yang and Yujia Zhou generated the data Shasha Tao and Guoyuan Lu supervised the data.

Figure 5:

In Figure 5, Jianzhong Li, Wangjianfei Yu and Youjing Yang generated the data , In Figure S5, Qianmin Li and Youjing Yang generated the data. Shasha Tao and Lei Shen supervised the data.

Figure 6:

In Figure 6 , Jianzhong Li and Youjing Yang generated the data and assembled the figure.
.

Signed for and on behalf of the Author(s):	Print Name:	Date:
Shasha Tao	Shasha Tao	2021-06-02
